# Technical limitations of REBOA in a patient with exsanguinating pelvic crush trauma: a case report

**DOI:** 10.1186/s13037-019-0204-6

**Published:** 2019-06-24

**Authors:** Orkun Özkurtul, Holger Staab, Georg Osterhoff, Benjamin Ondruschka, Andreas Höch, Christoph Josten, Johannes Karl Maria Fakler

**Affiliations:** 1 Department of Orthopedic, Trauma, and Plastic Surgery, University Hospital of Leipzig, Liebigstraße 20, 04103 Leipzig, Germany; 2 Department of Visceral, Transplantation, Thorax and Vascular Surgery, University Hospital of Leipzig, Liebigstraße 20, 04103 Leipzig, Germany; 3 Institute of Legal Medicine, Medical Faculty University of Leipzig, Johannisallee 28, 04103 Leipzig, Germany

## Abstract

**Background:**

Resuscitative endovascular balloon occlusion of the aorta (REBOA) is an effective adjunct in hemodynamic unstable patients with uncontrolled and non-compressible torso hemorrhage promoting temporary stability during injury repair. The aim of our study was to analyze real life usability of REBOA based on a case report and to review the literature with respect to its possibilities and limitations.

**Case presentation:**

We present the case of a 17-years old female patient who sustained a severe roll-over trauma and pelvic crush injury as a bicyclist by a truck. Upon arrival of the first responders, the patient was awake, alert, and following commands.

Subsequent to lifting the truck, the patient became hypotensive and required cardiopulmonary resuscitation, application of a pelvic binder, and endotracheal intubation at the accident scene. She was then admitted by ambulance to our trauma center under ongoing resuscitative measures. After primary survey, it was decided to perform a REBOA with surgical approach to the left femoral artery. Initial insertion of the catheter was successful but could not be advanced beyond the inguinal region. Hence, the patient was transferred to the operating room (OR) but died despite maximum therapy. In the OR and later autopsy, we found a long-distance ruptured and dehiscent external iliac artery with massive bleeding into the pelvis in the context of a bilateral vertical shear fractured pelvic bone.

**Conclusion:**

REBOA can be a useful adjunct but there is a major limitation with potential vascular injury after pelvic trauma. In these situations, cross-clamping the proximal aorta or pre-peritoneal pelvic packing as “traditional” approaches of hemorrhage control during resuscitation may be the most considerable methods for temporary stabilization in severely injured trauma patients. More clinical and cadaveric studies are needed to further understand indications and limitations of REBOA after severe pelvic trauma.

## Background

**Fig. 1:**
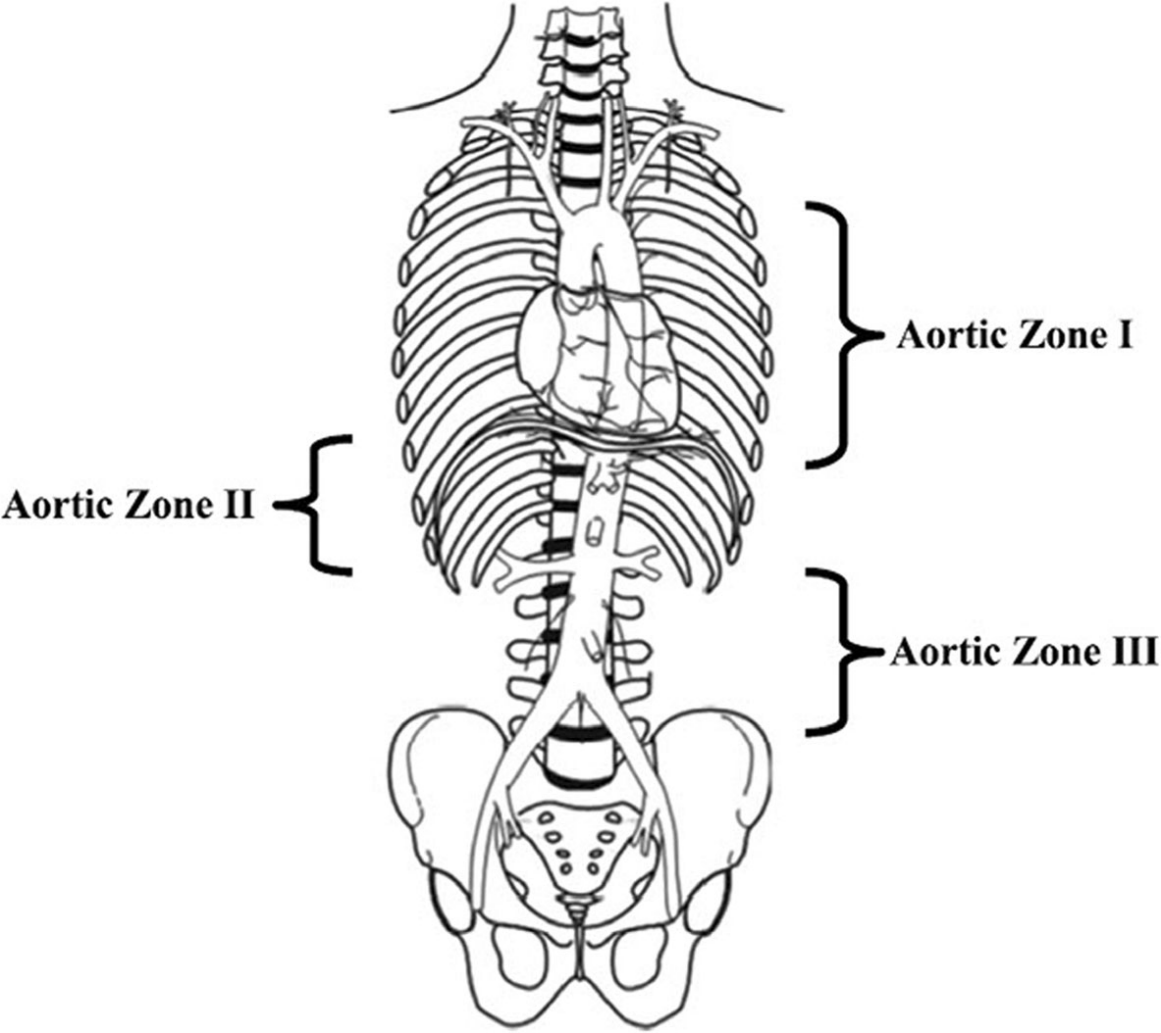
REBOA Zones reproduced with permission from Stannard et al. (Resuscitative Endovascular Balloon Occlusion of the Aorta (REBOA) as an Adjunct for Hemorrhagic Shock. The Journal of Trauma: Injury, Infection, and Critical Care. 1. Dezember 2011;71 [6]:1869–72)

Resuscitative endovascular balloon occlusion of the aorta (REBOA) is becoming increasingly common in patients with severe multiple injuries [1]. It is considered an effective adjunct in hemodynamically unstable patients with uncontrolled and non-compressible torso hemorrhage promoting temporary stability during injury repair. It is, hence, being widely discussed as a considerable alternative to emergency resuscitative thoracotomy, aortic cross-clamping and preperitoneal packing in severely injured patients [2–5]. The principle of REBOA was first described by Hughes in 1954 during the Korean War [6]. It is designed to stop the circulation and therefore the bleeding distal to the occluded area while sustaining sufficient circulation proximal to it [6–8]. That results in increased cardiac afterload and proximal aortic pressure and subsequent increase in myocardial and cerebral perfusion. It is more commonly used in non-trauma caused situations such as significant bleedings of the post-partum uterus, gastro-intestinal bleeding, in ruptured abdominal aortic aneurysms or exsanguination during pelvic surgery [7]. Although the exact indication for the use of REBOA has not yet been clarified and its handling is not fixed in guidelines yet, it is essentially thought to help in cases of acute shock due to massive hemorrhage regardless of trauma or non-trauma related causes [9]. Stannard et al. published an article describing a step-by-step approach of REBOA use in which three zones for balloon deployment are defined. Zone I extends from the origin of the left subclavian artery to the coeliac artery (approximately vessel diameter of 20 mm for young adult), Zone III extends from the lowest renal artery to the aortic bifurcation (Fig. 1) [8]. At first glance, the procedure appears less invasive through an arterial approach mostly using a transfemoral balloon catheter and relatively easy to perform, even for non-surgeons. But there are some severe complications and limitations such as the prolonged occlusion of the aorta that can lead to organ failure due to resulting ischemia-reperfusion injury distal of the occlusion, vessel injuries (aortic dissection, rupture, and perforation) or misplacement of the wire or the balloon within the arterial system [10–12]. Some authors support the broad application of this procedure in patients with profound shock due to exsanguination, some even in preclinical situations [7, 8, 13, 14]. Hemorrhage in patients with unstable high-energy pelvic fractures can be devastating and may be accessible to REBOA intervention. However, the potential of trauma-related concomitant injuries of the iliac arteries deserve special attention and has not yet been highlighted in case reports. Hence, we would like to present the case of a patient with hemorrhagic shock and a massive pelvic injury after a traffic accident.

## Case presentation

**Fig. 2:**
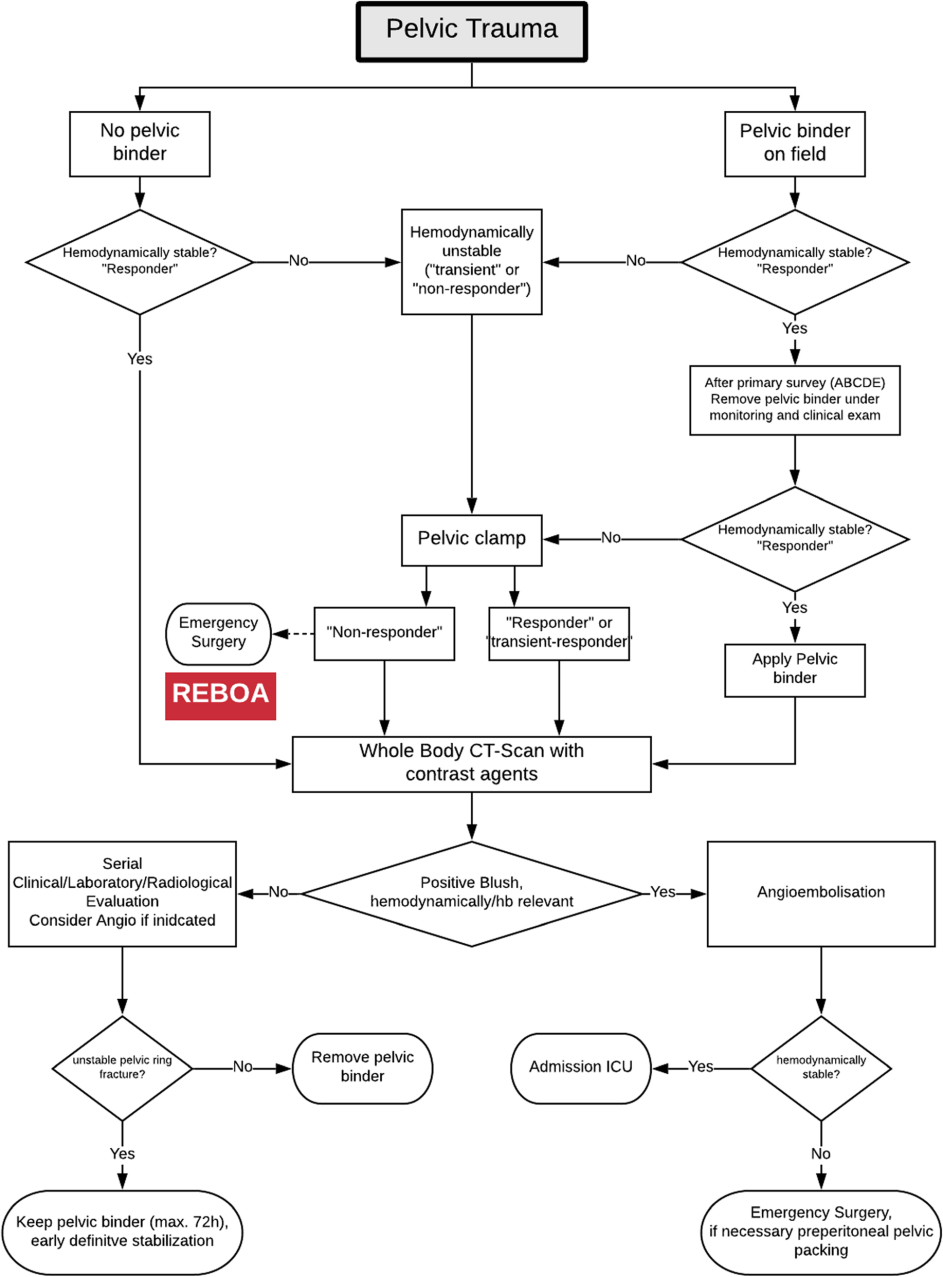
Leipzig Pelvic Trauma Algorithm in patients with hemorrhagic shock

**Fig. 3:**
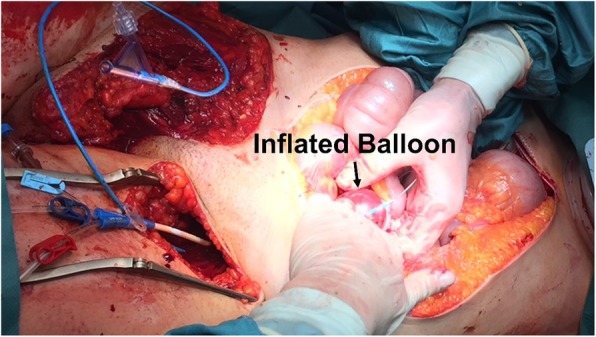
Loose catheter in the pelvis during emergency laparotomy in the operation room

**Fig. 4:**
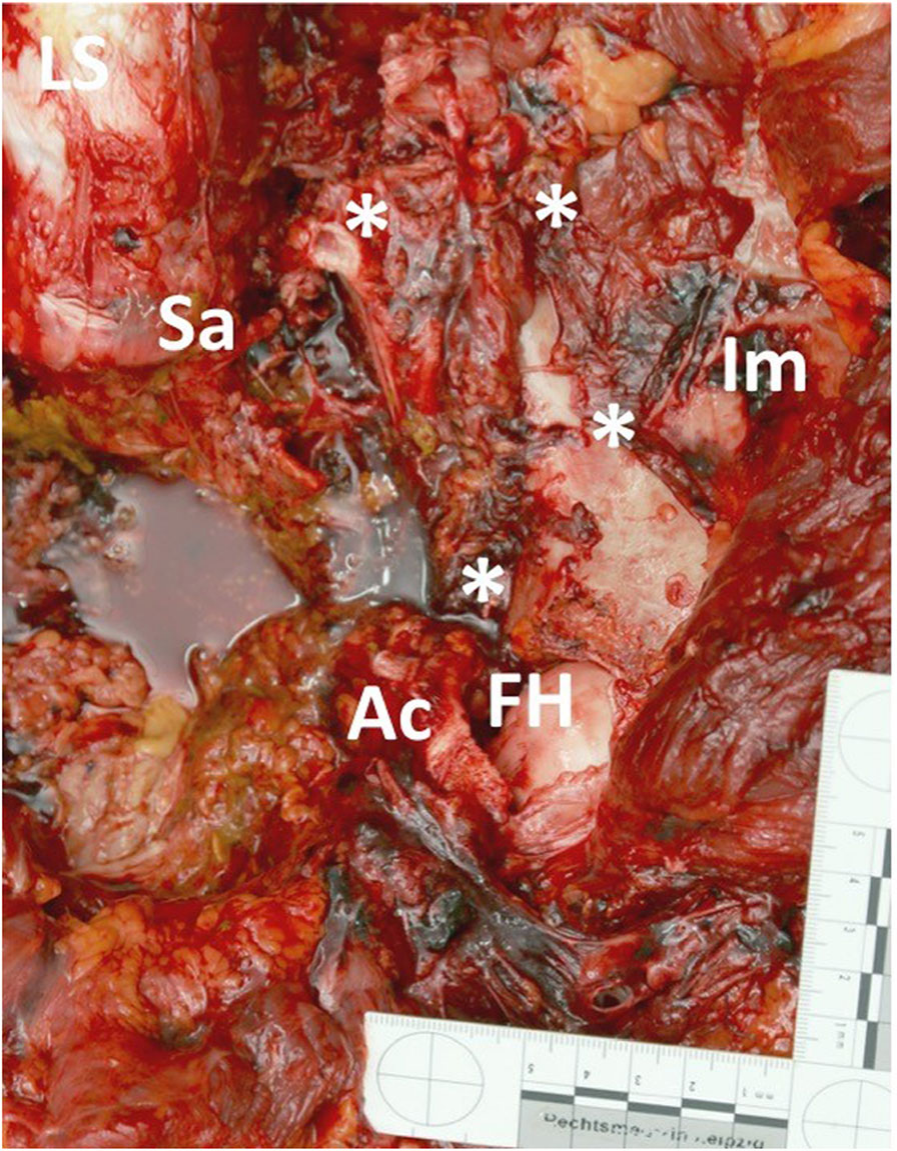
Acetabulum fracture with impacted femoral head left-sided. **LS**: Lumbar spine, **Sa**: Sacrum, **Im**: Ileum, **Ac**: Acetabulum, **FH**: Femur head, *: fracture lines

**Fig. 5:**
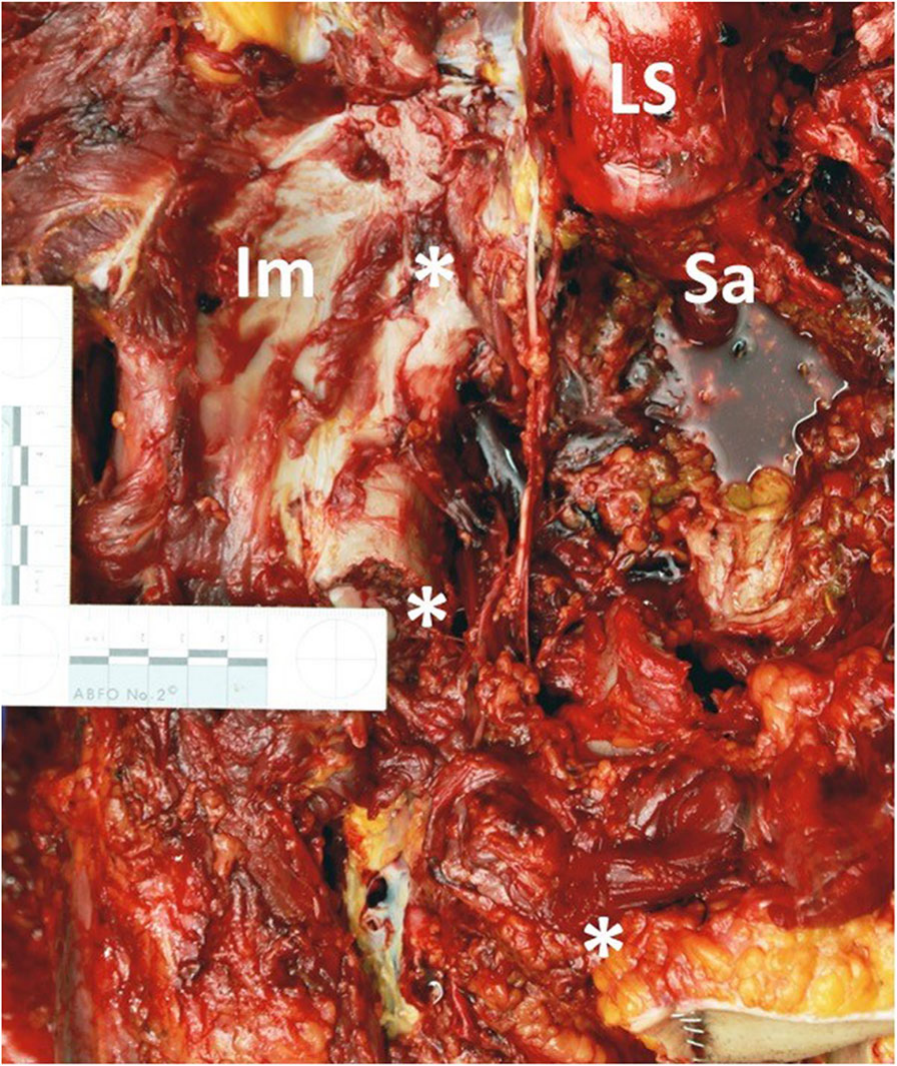
Fractured and unstable pelvis right-sided. **LS**: Lumbar spine, **Sa**: Sacrum, **Im**: Ileum, *: fracture lines

A 17-years old female patient sustained a severe roll-over trauma and pelvic crush injury as a bicyclist rolled over by a truck. Upon arrival of the first responders, the patient was awake, alert, and following commands. The rescue of the patient required lifting of the truck. The patient suddenly became unstable when the pressure of the tire on the pelvis decreased. Subsequent to lifting the truck, the patient became hypotensive and required cardiopulmonary resuscitation, application of a pelvic binder, and endotracheal intubation at the accident scene. She was then admitted by ambulance to our trauma center under ongoing resuscitative measures. However, no intravenous access was established yet, only an intraosseous needle was placed into the left proximal tibia. After primary survey following the principles of ATLS®, a pelvic C-clamp was applied and a massive transfusion protocol was activated. In total, 10 units of concentrated blood and 10 units of fresh frozen plasma were given. In case of non-responding situations our further approach is immediate emergency surgery (Fig. 2). REBOA is not an integral part of the algorithm in our institution yet. It was decided to perform a Venae sectio and to establish a REBOA with surgical approach to the left femoral artery. We use a conventional aortic stent graft balloon catheter (Reliant Stent Graft Balloon Catheter, Medtronic, Minneapolis, USA) as occlusion device. The initial insertion of the catheter was successful but could not be advanced beyond the inguinal region. Hence, the patient was transferred to the operating room (OR) but died despite maximum therapy before finalization of the surgical steps and after 100 min of ongoing cardiopulmonary resuscitation. In the OR, we carried out an emergency laparotomy with a standard midline approach and could find exsanguinating bleeding in the pelvis. Moreover, the reason for the missing sufficient catheter placement could be found (Fig. 3). The catheter had been inserted correctly into the femoral artery but then exited the vessel through a vascular injury of the iliac artery and, hence, came to its end in the inner pelvis. Later, a forensic autopsy was performed, showing a long-distance ruptured and dehiscent external iliac artery with massive bleeding into the pelvis, and a bilateral vertical shear fracture of the pelvis with retro- and preperitoneal hematoma. The acetabulum was fractured on both sides and the left femur head was impacted into the lesser pelvis. The fracture pattern was concluded to be a sufficient reason for the long distance ruptured iliac vessel ipsilaterally (Figs. 4 and 5). However, it was not possible to assess with certainty whether the vessel was already ruptured by the over-roll accident, or whether this injury has totally or partly been a complication related to the sheath insertion. Given the massive surrounding hemorrhage a vessel injury existing before circulation collapse and starting of the CPR was most plausible from a forensic point of view.

## Conclusion

Cross-clamping the proximal aorta or pre-peritoneal pelvic packing as “traditional” approaches for hemorrhage control during resuscitation are the most considerable methods for temporary stabilization in severely injured trauma patients and both methods are widely established during the past decades. REBOA can be a useful adjunct but there are some major limitations and the use should be limited and performed only by very experienced physicians or perhaps only by surgeons, who are aware of the potential access site consequences [15] and are rapidly able to start surgical rescue.

In our case it was necessary to prepare an extended surgical approach to the femoral artery due to the massive trauma damage of the pelvic region. Moreover, the catheter could not be advanced after inserting and inflation was therefore inadequate and could even be harmful. A surgical approach must be considered when assessment is not ensured. The in-hospital mortality rate of blunt trauma patients after REBOA in different studies varies from 28 to 76% versus a mortality rate of 62.5% [13] after resuscitative thoracotomy and aortic cross-clamping. A recent meta-analysis by Manzano-Nunes et al. analyzing groin access associated complications after the use of REBOA found an incidence of 4–5% [16]. The authors rightly conclude that the available data lacks at standardized protocols in handling and workflows. The same group compared REBOA with resuscitative thoracotomy in non-compressible torso hemorrhage patients in another study considering pelvic fracture management including the use of packing, external fixation, and surgery. The meta-analysis of unadjusted odds ratios in patients that underwent REBOA versus resuscitative thoracotomy showed that the odds of mortality were lower in the REBOA group [17]. In their analysis they underline the fact that resuscitative thoracotomy in most studies was performed in patients with a higher physiological exhaustion and with a lower probability of survival which illustrates a lack of concrete indications for REBOA use in trauma patients.

Some authors are convinced of the superior overall survival after REBOA compared to aortic cross-clamping in patients in profound shock [13]. In a recent meta-analysis addressing the use of REBOA in the management of major bleeding, 89 studies were included [7] and it was found that REBOA increases systolic blood pressure in hemorrhagic shock and is an adjunct for endovascular and open repair in hemodynamic instability with an iatrogenic related injury during vessel approach below 5% [7]. However, the studies mentioned differ fundamentally and there are only a few studies with standardized protocols while others are considerably affected by selection bias. With respect to aorta occlusion related iatrogenic injuries, rates between no injuries [18] and 28% [19] are reported.

In an autopsy study investigating potential REBOA usage in a post-mortem post-traumatic cohort, it could be shown that there are absolute contraindications even in patients in extremis e.g. with penetrating chest trauma [20]. In particular, the pelvic region deserves careful attention with respect to vessel injuries given its complex anatomical vascularity. It is well known that the venous sacral plexus is one major cause of hemorrhage death in patients suffering high energy pelvic disruption [21, 22]. Although plexus bleeding can be life-threatening, major vessel disruption as in the case presented more likely results from massive anterior-posterior compression injuries sometimes combined with impacted acetabular fracture, which is very rare [23]. In cases of complex pelvic trauma, arterial injuries occur in up to 20% of cases and venous bleeding from the presacral or perivesical venous plexus in 80% [22]. The commonly used transfemoral approach appears contraindicated considering our case and potential additional damage to the tissue and vessels. Thabouillot et al. performed a retrospective register study of trauma patients with bleeding of abdominal, pelvic and junctional origin with uncontrolled hemorrhagic shock and attempted resuscitation on scene [24]. They conclude that REBOA should be available on-scene and used by trained emergency personal. However, side effects are considerable high and can even be life-threating if the application is not safely or time is spent for inadequate attempts. Moreover, invasive methods for circulation observation and the temporary survival effect can lead to a higher rate of deaths if occlusion time is longer than 30 min [25]. For this reason, partial REBOA was developed to address and reduce ischemia-related metabolic and inflammatory risk [26]. Even though technical skills are manageable for non-surgeons, in our opinion the procedure must not be seen as an individual concept but must be able to provide the complete repertoire of operative interventions without delay. Specific problems during balloon occlusion are accessing the wrong vascular tree, misplacement of the wire or balloon within the arterial system, the creation of dissection flaps or other arterial injury, retroperitoneal hemorrhage, the development of lactic acidosis and organ dysfunction, and the development of clots which may lead to limb ischemia [25]. These complications related to vessel injuries during insertion are well described in the vascular literature [27, 28], but the majority of these studies investigates elective interventions and comparison is not adequate to high risk situations such as CPR after trauma. In addition, there are case reports describing a worsening of the hemorrhage situation with massive intracranial hemorrhage after application of REBOA [12, 29], most likely because of its consequential increased cerebral perfusion.

It is the authors’ opinion that the REBOA application is a helpful adjunct, but in the majority of studies a less critical consideration of possible side effects has to be observed. The emergency thoracotomy and laparotomy with or without aortic cross clamping are still important repertoires in cases of non-compressible hemorrhages. REBOA should be trained and performed by an acute care surgeon or an interventionalist (vascular surgeon or interventional radiologist) and in order to resolve possible vascular complications, a vascular surgeon must be available within reach. Further studies are needed to understand the physiological effects, indication of REBOA and mostly due to lack of studies the evaluation of contraindications in case of major pelvic trauma.

In summary, REBOA is an important, less invasive and effective tool for hemorrhage control in non-compressible bleedings, with the ability to place the catheter at the level of intended occlusion, the opportunity to monitor intra-aortic pressure, with presumptive at least similar overall survival compared to rescue thoracotomy and aortic cross-clamping. However, in patients with unstable pelvic fractures, potential ilio-femoral vascular injuries pose a contraindication for REBOA and standard open procedures should be preferred.

Further studies with more comparable patient population and special consideration of pelvic trauma mechanisms and subsequent injuries are needed to define the fields of application of REBOA in detail. However, special attention has to be paid in cases of lethal outcome after major trauma with pelvic injuries and such fatalities should consequently be ordered for forensic autopsies.

## Data Availability

The dataset used and analyzed during the current study is available from the corresponding author on reasonable request.

## Abbreviations

AIS: Abbreviated Injury Score
ATLS: Advanced Trauma Life Support
ED: Emergency department
GCS: Glasgow Come Scale
ISS: Injury Severity Score
OR: Operation room
OST: On-scene time
REBOA: Resuscitative endovascular balloon occlusion of the aorta
